# Personal-MetaboHealth, an actionable health check in middle age, is improved by an effective lifestyle intervention in those at risk

**DOI:** 10.1186/s12916-026-05178-z

**Published:** 2026-09-07

**Authors:** Niels van den Berg, Gabriela Natalle Lopes, Fatih A. Bogaards, Marian Beekman, Edson Amaro Junior, P. Eline Slagboom, Joris Deelen

**Affiliations:** 1 Section of Molecular Epidemiology, Department of Biomedical Data Sciences, Leiden University Medical Center, Leiden, The Netherlands; 2 Health Campus The Hague, Leiden University and Leiden University Medical Center, The Hague, The Netherlands; 3 Global Advanced Technologies for Equity, Hospital Israelita Albert Einstein, São Paulo, Brazil

**Keywords:** Personal-MetaboHealth, MetaboHealth, Cardiometabolic health, Biomarkers, Lifestyle intervention, Ageing

## Abstract

**Background:**

MetaboHealth represents a novel indicator of overall health in middle age and may potentially be suitable as actionable health check in prevention strategies. MetaboHealth is a blood-based metabolomic composite score that predicts a wide range of age-related conditions and mortality in large European cohorts.

**Methods:**

Here, we investigated whether MetaboHealth can be personalised and limited to clinically validated, i.e. CE-marked, metabolic biomarkers. Next, we assessed whether the updated MetaboHealth score predicts all-cause mortality and cardiometabolic disease incidence and can be improved by a lifestyle intervention. To personalise MetaboHealth, we scaled the metabolic biomarkers using a Dutch reference population (i.e. the Biobanking and BioMolecular Research Infrastructure Netherlands) and, in addition, based the score solely on clinically validated metabolic biomarkers.

**Results:**

The novel version of the score, Personal-MetaboHealth, retained predictive accuracy for all-cause mortality and showed an even stronger association with incident cardiometabolic disease in the Leiden Longevity Study (LLS) in which 2,404 participants were followed for up to 22 and 16 years for mortality and morbidity, respectively. The association of Personal-MetaboHealth with all-cause mortality remained robust after adjusting for smoking quantity, alcohol consumption, and medication use, while the cardiometabolic disease association was attenuated due to smoking quantity. Each unit increase in Personal-MetaboHealth was associated with a 13.69 year later onset of the first cardiometabolic disease in the LLS. Next, we showed that Personal-MetaboHealth can be improved by a 3-month combined lifestyle intervention in middle aged individuals (Growing Old Together study), specifically in those at risk, i.e. with an unhealthy score at baseline.

**Conclusions:**

Personal-MetaboHealth thus offers a potential actionable health check in middle age for early prevention and extension of healthy lifespan.

## Background

The global population is undergoing a demographic shift towards a high proportion of older age groups, such that nearly one in five persons will be aged 60 years or older by 2030 [1]. In parallel to this demographic shift, our lifespan continues to increase while gains in years lived free of disease have been more limited, resulting in prolonged morbidity and disability. The co-occurrence of this demographic shift and slower gains in disease-free life years places a significant burden on healthcare systems and results in high costs [2], underscoring the importance to improve quality of life and extend the years lived in good health [3]. Therefore, preventive strategies must move beyond symptom-based detection and instead focus on the early identification of individuals at increased risk of age-related conditions (e.g. decline of cognitive, metabolic, and muscle function), diseases, and multi-morbidity, while clinical symptoms have not manifested yet. Biomarkers allowing such early identification are necessary to professionally support evidence-based lifestyle programs.

Besides a diagnosis as wake-up call for a lifestyle change, affordable, scalable, and validated molecular biomarkers may serve as motivational tools for those at risk in the prevention of overall health decline in the population [4, 5]. Recent technological advances, such as standardised omics-based measurements, offer novel opportunities to generate biomarkers that capture age-related systemic changes that precede disease onset, opening a window of opportunity for prevention [6]. Although many overall health scores, aiming to capture ageing-related decline, have been constructed, most are based on cross-sectional data or are expensive to generate [6]. For this reason, the MetaboHealth score was developed as a cost-efficient and scalable high-throughput nuclear magnetic resonance (¹H-NMR) spectroscopy measurement-based biomarker indicating overall health. MetaboHealth was trained on prospective mortality data from 12 large cohorts and was generated to predict mortality risk in population-based cohorts [7], which it does at all ages, but particularly successful in the middle-aged population. Beyond accurately predicting mortality risk, an increase in MetaboHealth is associated with a wide range of age-related conditions and functional decline, including increased somatic frailty, worse COVID-19-related outcomes, decreased cognitive processing, poorer memory, and reduced functional independence [8–11]. Together, these findings established MetaboHealth as a valuable tool for assessing population-level differences in overall health and a potential health check for prevention through lifestyle programs.

However, to bring omics-based overall health scores into clinical application several innovations are needed, which we aimed to address in the current study. First, applications of general health scores, including MetaboHealth, have been limited to cohort-level application as they usually require within-cohort scaling [12]. Hence, their potential utility in providing individualised health estimates remains unexplored. Second, while MetaboHealth has been associated with incidence and prevalent morbidity in the UK Biobank [13], it remains unknown whether it also captures time to first chronic disease onset. Establishing this link is critical to assess whether predictors of mortality extend to morbidity, which together define the principal outcomes of ageing-related health. Third, a molecular marker potentially supporting stratification of middle-aged individuals at risk of rapid overall health decline would ideally be actionable, i.e. showing a beneficial change in response to health-improving clinical or lifestyle interventions [14].

To address these points, we used data from the Leiden Longevity Study (LLS), a three-generation (F0-2) cohort established in 2002 [15]. We focused on the second generation (F2, *N* = 2,404) of LLS participants, in which Nightingale Health metabolomics data as well as long-term morbidity (up to 16 years) and mortality follow-up (up to 22 years) were collected. First, we investigated the MetaboHealth score’s potential for individualised application by constructing Rescaled-MetaboHealth [16]. Next, we constructed Personal-MetaboHealth using the subset of clinically validated metabolic biomarkers i.e. biomarkers validated by Nightingale Health for use in an in vitro diagnostic medical device (CE-marked) [14]. We subsequently determined the associations of the different MetaboHealth scores with all-cause mortality and examined whether the scores predicted age at first cardiometabolic disease onset. Finally, using data from the Growing Old Together (GOTO) study [17] we investigated to what extent a short term (3-month) combined lifestyle intervention [17] changed the Personal-MetaboHealth score in individuals aged >60 years at risk of rapid overall health decline according to their baseline score.

## Methods

### Study populations

#### Leiden longevity study

The LLS, initiated in 2002 in the Netherlands, is an ongoing cohort designed to investigate the molecular and environmental factors that may contribute to longevity [15]. The study recruited long-lived siblings, men aged 89 years or older and women aged 91 years or older (F1), together with their offspring and the offspring’s partners (F2), resulting in a cohort of 421 long-lived families. We also collected demographic data of the parents of the F1 generation (i.e. F0). Here, we focused specifically on the F2 generation. By 2018, 246 (out of 2,404) participants had died, resulting in 10.2% of the cohort being lost before morbidity follow-up ended. By 2025, 489 participants (20.3%) had died, while 498 (20.7%) had developed morbidity.

The cohort protocol was approved by the Medical Ethical Committee of the Leiden University Medical Center at the 16^th^ of August 2002, before the start of the study (P01.113), when the first participant enrolled on the 5^th^ of September 2002. In accordance with the Declaration of Helsinki, the LLS obtained informed consent from all participants prior to entering the study.

#### Growing old together study

The GOTO study was a 13-week non-randomized, non-controlled, single-arm, open-label lifestyle intervention trial including 164 healthy older adults (83 males, 81 females, mean age 62.8  ±  5.7 years, mean BMI 26.9  ±  2.5 kg/m²). Participants underwent a 25% reduction in energy balance, achieved through a combination of a 12.5% decrease in caloric intake and a 12.5% increase in physical activity, as described by van de Rest and colleagues [17]. Prior to the intervention, baseline energy intake and expenditure were assessed using a 150-item food frequency questionnaire and the International Physical Activity Questionnaire - Short Form. Personalized intervention guidelines were developed by a dietician and a physiotherapist in consultation with the participants, taking into account individual preferences and physical abilities. Dietary recommendations were designed to align as closely as possible with the Dutch Guidelines for a Healthy Diet. To promote adherence, participants were encouraged to increase physical activity in ways that fit their daily routines, with the aim of achieving full integration into regular daily life both during and after the intervention. Physical activities typically included walking, cycling, household tasks, and participation in local sports activities, either individually or with a partner. As described by Bogaards and colleagues, the activity and dietary compliance was self-reported during the GOTO intervention and 153 participants were considered compliant to the intervention program [18]. Out of these 153 participants, the Personal-MetaboHealth score was available in 135 participants. The primary outcome of the GOTO study was a significant reduction in fasting insulin levels, as previously reported [17].

The GOTO study protocol was approved by the Medical Ethical Committee of the Leiden University Medical Center before the start of the trial (P11.187). In accordance with the Declaration of Helsinki, the GOTO study obtained informed consent from all participants prior to entering the trial. This trial was registered at the Dutch Trial Register (https://onderzoekmetmensen.nl/en/trial/27183) as GOT NL3301 and can also be found at the international clinical trials registry platform as NL-OMON27183. The first participant was enrolled on the 11^th^ of June 2012, while the last participant was enrolled on the 17^th^ of January 2013.

#### Biobanking and BioMolecular Research Infrastructure Netherlands

Another resource included in this project is the Biobanking and BioMolecular Research Infrastructure Netherlands (BBMRI-NL), a nationwide initiative that connects Dutch biobanks and cohort studies to support large-scale biomedical research. Established in 2009 [16], it combines biological samples and data from a wide range of cohorts: ALPHAOMEGA, BIOMARCS, CHARM, CHECK, DMS, DZS_WF, ERF, FUNCTGENOMICS, GARP, HELIUS, HOF, LIFELINES, LLS_PARTOFFS, LLS_SIBS, MRS, NESDA, PROSPER, RS, STABILITEIT, STEMI_GIPS-III, TOMAAT, UCORBIO, VUMC_ADC. Study information, including the ages at which biomaterial was obtained, is provided in our earlier work [19] and by the BBMRI-NL consortium [16], including omics datasets such as metabolomics. All participating biobanks operate under ethical approval of their respective institutions, with informed consent obtained from all participants.

### Mortality data

Since the start of recruitment of the LLS, F2 generation mortality data has been updated through the Dutch Personal Records Database, a Dutch governmental service for identity information which is part of the nationwide Dutch population registry system [20]. This approach guarantees a consistent and reliable determination of vital status of the LLS participants.

### Morbidity data

Morbidity data were available for a subset of LLS participants (*N* = 1,347, 56%) and were provided by the general practitioners of the F2 generation participants. The morbidity data covers each participant’s disease history from birth until 2018, with general practitioners extracting information on age-related cardiometabolic diseases and the year of diagnosis directly from their health records. The records are routinely updated, even when individuals change doctors, ensuring continuity of information.

For the current study, cardiometabolic diseases reported in the questionnaires were defined according to the International Statistical Classification of Diseases and Related Health Problems 10th Revision. The codes included were stroke (I64), cerebrovascular accident (I63), angina pectoris (I20), acute myocardial infarction (I21), essential (primary) hypertension (I10), and diabetes mellitus (E10–E14).

### Questionnaire and pharmacy data

Information on smoking and drinking was obtained via a questionnaire administered during the entry phase of the study, while medication data were acquired directly from participants’ pharmacies. To calculate smoking quantity, we multiplied the number of cigarettes per day by 365.25 and by the number of years smoked and divided this number by 10, similar to what was done by Kuiper and colleagues [14]. Alcohol consumption was measured in glasses per day using a food frequency questionnaire, in which intake was quantified in standard units corresponding to 10 grams of alcohol per unit.

### Metabolomics measurement and construction of MetaboHealth scores

We used metabolomics data derived from EDTA plasma or serum samples collected at the time of recruitment of the study participants (LLS and BBMRI-NL) or at the baseline and end of the intervention (GOTO study). All samples were quantified with the Nightingale Health platform using ¹H-NMR spectroscopy following the company’s standard experimental procedures, as previously described [4, 11, 12], and we used the data from the re-quantified version released in 2020 [21].

We constructed three different MetaboHealth scores. First, the original MetaboHealth score, hereafter referred to as MetaboHealth, which includes the 14 metabolic biomarkers acetoacetate (AcAce), albumin (Alb), glucose (Glc), glycoprotein acetyls (Gp), histidine (His), isoleucine (Ile), lactate (Lac), leucine (Leu), phenylalanine (Phe), the ratio of polyunsaturated fatty acids to total fatty acids (PUFA/FA), total lipids in small HDL (S-HDL-L), valine (Val), mean diameter for VLDL particles (VLDL-D), and total lipids in chylomicrons and extremely large VLDL (XXL-VLDL-L). Given MetaboHealth was trained as an indicator of prospective mortality, higher levels indicate an increased mortality and age-related disease risk. Since we instead want to use MetaboHealth as a health indicator, we calculated its inverse so that increasing levels indicate increased survival and health. To enable application at the individual level, we subsequently rescaled the MetaboHealth score using metabolic biomarker reference distributions from BBMRI-NL, thereby making the score representative of the Dutch population, but with a strong, although not exclusive, Caucasian focus. We termed this score Rescaled-MetaboHealth. Finally, we constructed a clinically validated individualised MetaboHealth score based on the 10 metabolites that received CE marking (i.e. PUFA/FA, Glc, Alb, Gp, Phe, Ile, Leu, Val, His, and Lac). This clinically validated individualised MetaboHealth score is hereafter referred to as Personal-MetaboHealth. To improve interpretability, we rescaled all MetaboHealth scores in a way that a higher score indicates better health.

MetaboHealth is calculated as follows:

1 $${\rm{MetaboHealth}}_{\rm{i}}=\frac{{\sum }_{m=1}^{14}{\beta }_{m}{\rm{\cdot }}Z(\log ({{{X}}}_{{{im}}}))-{\mu }_{{{LLS}}}}{{{\rm{\sigma }}}_{{LLS}}}$$

2 $$Z(\log \left({X}_{{im}}\right))=\frac{\log \left({X}_{{im}}\right)-{\mu }_{m,{LLS}}}{{{\rm{\sigma }}}_{m,{LLS}}}$$

Where ${X}_{{im}}$ denotes the raw concentration of metabolite m for individual i. $\log ({X}_{{im}})$ denotes the log-transformed metabolite value. $Z(\log \left({X}_{{im}}\right))$ denotes the z-scaling of the log-transformed metabolite m using the metabolite mean and standard deviation from the LLS as illustrated in expression 2. ${\beta }_{m}$ represents the regression coefficient corresponding to metabolite m. These regression coefficients are obtained from the original MetaboHealth paper [7]. The weighted metabolite sum score MetaboHealth is subsequently standardized again using the mean (${\mu }_{{LLS}}$) and standard deviation (${\sigma }_{{LLS}}$) of the weighted sum score in the LLS dataset. The final score therefore represents a z-standardized weighted metabolite profile relative to the LLS reference population.

Rescaled-MetaboHealth follows expression 1 but all z-scaling parameters in expression 2 based on the ${LLS}$ are now based on BBMRI-NL. This effectively means that the “LLS” parts in expression 2 are replaced with “BBMRI-NL”. Personal-MetaboHealth follows the same adaptation to expression one but instead of using 14 metabolites now only the 10 clinically validated metabolites are used. In effect, this means the ${\sum }_{m=1}^{14}$ is replaced by ${\sum }_{m=1}^{10}$. To further facilitate clinical application Personal MetaboHealth was also scaled using the BBMRI-NL data. This effectively means that the “LLS” parts in expression 1 are replaced with “BBMRI-NL”. Z-scaling of the scores in the LLS was applied to bring them into the same distribution and unit, thereby enabling direct comparison of effect sizes across different analyses. In contrast, scaling Personal-MetaboHealth on BBMRI-NL was applied to have a well-defined zero value that represents the average score in the general Dutch population as defined using BBMRI-NL.

The script used to calculate the MetaboHealth scores from raw Nightingale Health metabolomics data is provided as free, open-access material at https://git.lumc.nl/publications/individualized-metabohealth.

### Statistical analysis

All statistical analyses were performed with R (version 4.3.2). A p-value threshold of <0.05 was considered statistically significant and results are presented with corresponding 95% confidence intervals (CIs).

Our initial analyses were focused on the F2 generation of the LLS. As a first analytical step, we used Cox random-effects (frailty) survival models to examine the extent to which the scores were associated with prospective mortality and first morbidity. These analyses were adjusted for sex and age at inclusion, with age at inclusion included as a left-truncation point in the survival analyses. We subsequently repeated these analyses with additional adjustment for smoking quantity, alcohol consumption, and medication use.

To estimate how the Personal-MetaboHealth score associates with time to first morbidity on the age scale, we used accelerated failure time (AFT) models. Unlike Cox proportional hazards models, AFT models directly quantify how covariates accelerate or decelerate the expected time until an event. This allows results to be interpreted in terms of the original time to event scale. In our analyses, time to event was defined as the difference between age at study entry and age at first disease or last follow-up. The AFT models were adjustment for sex, age at inclusion, and familial clustering. Several parametric distributions were tested (i.e. exponential, Weibull, Gaussian, logistic, log-logistic, and log-normal) and model fit was evaluated using Akaike and Bayesian information criteria. The exponential distribution provided the best fit. More details on how AFT models work, compare to Cox proportional hazards models, and the model specifications are provided in Additional file 1: Supplementary Methods.

Finally, we used the GOTO study to investigate whether, and to what extent, the Personal-MetaboHealth score improves following a combined lifestyle intervention. To assess the impact of the intervention on overall health, we analysed the changes in Personal-MetaboHealth using a linear mixed model adjusted for age and sex as fixed effects and family-id and person-id as random effects. The same linear mixed models were used to study the immune-metabolic health changes represented by blood pressure (systolic blood pressure and diastolic blood pressure), body composition (body mass index, waist circumference, total body fat %, and trunk fat %), serum markers of fat metabolism (fasting levels of LDL cholesterol, HDL cholesterol, triglycerides, and HDL cholesterol size), glucose metabolism (fasting insulin), and inflammation (fasting C-reactive protein (CRP)).

## Results

For the present study, we focused on the F2 generation of the LLS, consisting of 1,666 offspring and 738 partners, leading to a grand total of 2,404 participants. At baseline, participants had a mean age of 59.2 years and were followed for up to 22 years for all-cause mortality and 16 years for cardiometabolic disease incidence (Table 1).

**Table 1:** Demographic characteristics from the F2 participants of the LLS

Characteristics	
Participants, *N*	2,404
Offspring/partners, *N*	1,666/738
Age at inclusion, mean (SD)	59.1 (6.8)
Female, *N* (%)	1,326 (55.2)
Follow-up time mortality, mean (SD)	18.1 (3.9)
Deceased, *N* (%)	489 (20.3)
Age at death, mean (SD)	76.3 (8.6)
Disease free at inclusion, *N* (%)	1,403 (58.4)
Follow-up time cardiometabolic disease, mean (SD)	10.1 (3.7)
Incident cardiometabolic disease, *N* (%)	433 (30.9)
Metabolomics data available, *N* (%)	2,301 (95.7)
Smoking, *N* (%)	1,316 (54.7)
Smoking quantity (per 10 cigarettes a day), mean (SD)	8,015.6 (11,957.1)
Alcohol consumption (standardised glasses per day), mean (SD)	14.6 (14.8)
Medication use, *N* (%)	507 (21.1)

### The different MetaboHealth scores associate equally well with all-cause mortality

**Fig. 1:**
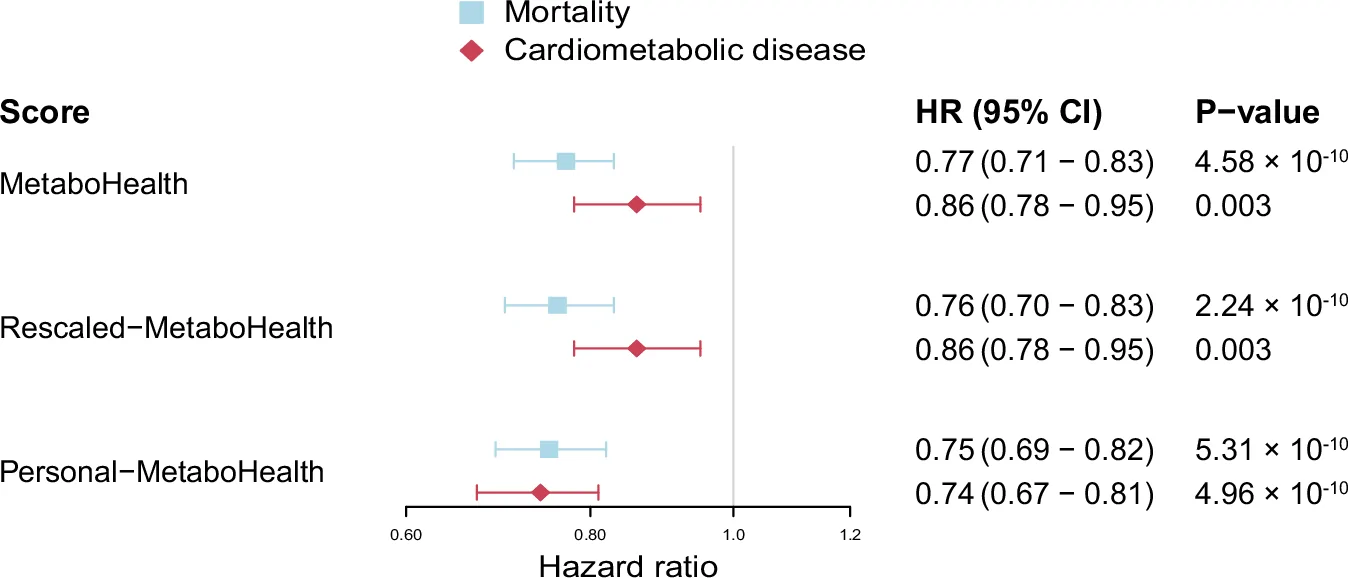
Association between the different MetaboHealth scores and all-cause mortality and cardiometabolic disease morbidity. The figure depicts the different MetaboHealth scores in association with time to mortality (light blue squares) and time to first cardiometabolic disease (red diamonds). The statistical models are adjusted for age, sex, and family relations using a Gaussian random effect. HR = hazard ratio; CI = confidence interval.

A key aim of this study was to evaluate whether our original MetaboHealth score could be adapted to estimate health status at the individual level. To this end, we compared three versions of the score, including the original one. As a first step, we compared the association of the different versions of the MetaboHealth score with prospective mortality. Our results indicate replication of our previous work, as we observed a strong association of the original MetaboHealth score with all-cause mortality in the LLS [7]. We showed that each standard deviation increase in the score corresponded to a 23% lower risk of death (Hazard Ratio (HR) = 0.77, 95% CI: 0.71–0.83, P = 4.58 × 10^-10^, Fig. 1). The mortality associations of the Rescaled-MetaboHealth (HR = 0.76, 95% CI: 0.70–0.83, P = 2.24 × 10^-10^, Fig. 1) and Personal-MetaboHealth scores (HR = 0.75, 95% CI: 0.69–0.82, P = 5.31 × 10^-10^, Fig. 1) were of similar strength, indicating that the predictive performance was preserved when the score was standardised to BBMRI-NL data and confined to the clinically validated subset of the original variables.

### Personal-MetaboHealth is most strongly associated with cardiometabolic disease onset

As a second step, we compared the association of the MetaboHealth scores with the onset of first cardiometabolic disease. To this end, we used the 1,403 participants from the LLS who were free of cardiometabolic disease at baseline. We observed that higher levels of all three MetaboHealth scores were strongly associated with a delayed onset of first cardiometabolic disease (Fig. 1). Notably, the strongest effect was observed for Personal-MetaboHealth, which showed a 26% decreased yearly risk of developing a first cardiometabolic disease with each increase in standard deviation (HR = 0.74, 95% CI: 0.67–0.81, P = 4.96 × 10⁻^10^). The effect of the Personal-MetaboHealth score was 12% stronger (HR = 0.74 vs. HR = 0.86) compared to the other two MetaboHealth scores.

### Adjustment for covariates attenuates the effects of the MetaboHealth scores

Given that previous studies have shown a strong association of the original MetaboHealth score with lifestyle factors [22–24], we performed robustness checks in which we adjusted for smoking quantity, alcohol consumption, and medication use. For all MetaboHealth scores the strength of the association with all-cause mortality was slightly attenuated, but remained statistically significant (P ≤ 0.006, Table 2). For cardiometabolic disease incidence, however, the associations of both the original MetaboHealth and Rescaled-MetaboHealth scores were no longer significant (P = 0.079 and P = 0.073, respectively), while the association of the Personal-MetaboHealth score was also attenuated (HR_Adjusted_ = 0.78 vs HR_Unadjusted_ = 0.74), but remained significant (P = 1.92 × 10^−4^, Table 2). Sensitivity analyses showed that the majority of the reduction in the association between the MetaboHealth scores and all-cause mortality and cardiometabolic disease incidence is caused by adjustment for smoking quantity (Additional file 1**:** Table S1).

**Table 2:** Association between the different MetaboHealth scores and all-cause mortality and cardiometabolic disease incidence

	HR (95% CI)	P-value	HR (95% CI)	P-value
**Mortality**	**Basic (** ***N*** ** = 2,301)**		**Adjusted (** ***N*** ** = 1,228)**	
MetaboHealth	0.77 (0.71 - 0.83)	4.58 ×10^−10^	0.81 (0.70 - 0.93)	0.004
Rescaled-MetaboHealth	0.76 (0.70 - 0.83)	2.24 ×10^−10^	0.79 (0.69 - 0.92)	0.002
Personal-MetaboHealth	0.75 (0.69 - 0.82)	5.31 ×10^−10^	0.82 (0.71 - 0.94)	0.006
**Cardiometabolic disease**	**Basic (*N*** = **1,347)**		**Adjusted (*N*** = **812)**	
MetaboHealth	0.86 (0.78 - 0.95)	0.003	0.89 (0.78 - 1.01)	0.079
Rescaled-MetaboHealth	0.86 (0.78 - 0.95)	0.003	0.89 (0.78 - 1.02)	0.073
Personal-MetaboHealth	0.74 (0.67 - 0.81)	4.96 ×10^−10^	0.78 (0.68 - 0.89)	1.92 ×10^−4^

HR, hazard ratio; CI, confidence interval. The basic models are adjusted for age at inclusion, sex, and family relations (random effect), while the adjusted models are additionally adjusted for smoking quantity, alcohol consumption, and medication use

### One unit increase in Personal-MetaboHealth delays cardiometabolic disease onset by over a decade

Instead of quantifying the effect of a marker on an outcome as a hazard ratio, it is also possible to quantify this at the age scale, i.e. the number of years of later/earlier onset of an outcome, using AFT models, which allows more intuitive interpretation. When applied to Personal-MetaboHealth, the AFT model showed a ‘time ratio’ of 1.36 (95% CI: 1.23–1.47; P = 2.66 × 10⁻^10^), indicating that each unit increase in Personal-MetaboHealth was associated with a 36% delay in the onset of first cardiometabolic disease. For an average participant in the LLS dataset, this corresponded to an approximately 13.69-year longer predicted median time until onset of the first cardiometabolic disease.

### Personal-MetaboHealth positively responds to a lifestyle intervention

For implementation of a biomarker as a health check in population health, it needs to be actionable, i.e. modifiable through an evidence-based intervention. We therefore investigated to what extent a 3-month combined lifestyle intervention (GOTO study) changed the Personal-MetaboHealth score in middle aged individuals (62.8 years (SD 5.7)). When we combined the data from all individuals within the study, we observed no change in the Personal-MetaboHealth score due to the intervention (β = 0.02, 95% CI: -0.07–0.11, P-value = 0.666).

Using the BBMRI-NL–scaled Personal-MetaboHealth score, GOTO study participants with scores below zero can be considered less healthy than the average Dutch population and thus represent a relevant target group for lifestyle interventions. This aligns with our previous finding that, despite the overall good health of the cohort, the intervention was most effective in improving health-related outcomes, such as systolic blood pressure, among participants who were relatively unhealthy at baseline [17]. We therefore stratified the GOTO study participants into two groups ( < 0, ‘unhealthy’ (*N* = 52) and ≥0, ‘healthy’ (*N* = 83)) based on the Personal-MetaboHealth scores at the GOTO study baseline measurement (Additional file 1**:** Fig. S1). The two groups showed no difference in age at baseline (P-value = 0.502) or sex distribution (P-value = 0.913, Additional file 1**:** Table S2).

**Fig. 2:**
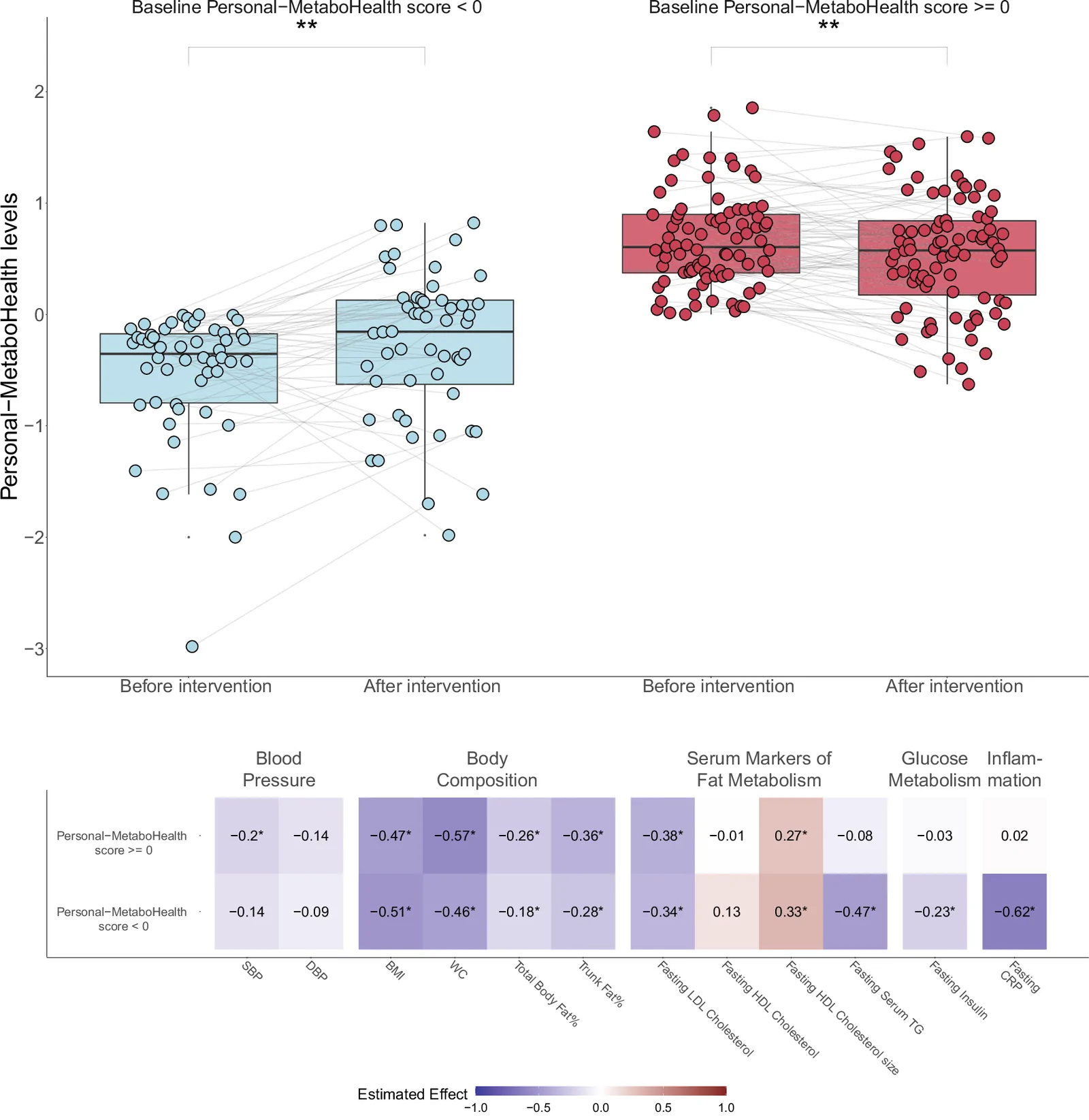
Changes in Personal-MetaboHealth, stratified by baseline scores. The figure depicts the change in Personal-MetaboHealth in the GOTO study for those with a baseline Personal-MetaboHealth below zero (top left) and for those with a Personal-MetaboHealth above or equal to zero (top right). Analyses were done using a mixed model with a Gaussian random effect to model individual changes over time. The analyses are adjusted for age at inclusion and sex as fixed effects (before intervention) and family-id and person-id as random effects. The significance level is represented by an asterisk: ** p-value < 0.01. The bottom part shows the change in the z-scaled health markers due to the intervention for participants stratified at baseline (above and below Personal MetaboHealth of 0). These analyses are also adjusted for age at inclusion and sex as fixed effects (before intervention) and family-id and person-id as random effects. Blue represents a decrease in the health marker due to the intervention, while red represents an increase. The strength of the colour indicates the effect size. An asterisk indicates that the association is significant (P < 0.05) after Benjamini-Hochberg correction for multiple testing. The unscaled changes in the health markers are depicted in Additional file 1: Table S2

We observed that the individuals in the unhealthy group significantly improved their score as a results of the intervention (β = 0.26, 95% CI: 0.10–0.42, P-value = 0.003), while the opposite was observed, although to a lesser extent, for the individuals in the healthy group (β = -0.13, 95% CI: -0.22–-0.04, P-value = 0.007; Additional file 1**:** Table S3). The metabolic biomarkers that seem to drive the increase in Personal-MetaboHealth in the unhealthy group are GlycA, Glc, Phe, Ile, and Leu, while in the healthy group the decrease in the score seems to be driven by changes in Alb, Phe, and His (Additional file 1**:** Table S3). In addition, we observed a difference between the two groups in other health-related parameters in response to the intervention, such as fasting triglyceride, CRP, and insulin levels (the primary outcome of the GOTO study). Markers of body composition and blood pressure showed a comparable change in both groups (Fig. 2, Additional file 1**:** Table S2), indicating that the Personal-MetaboHealth score is likely an actionable biomarker that can be altered through a lifestyle intervention in middle-aged at-risk individuals.

## Discussion

In this study, we investigated whether the MetaboHealth score, a biomarker of overall health, could be modified so it can be used as an actionable health check in middle age for prevention strategies. We created different versions of MetaboHealth, one that is individualised by scaling based on the Dutch population (Rescaled-MetaboHealth) and one that is additionally confined to the subset of clinically validated metabolic biomarkers (Personal-MetaboHealth). We subsequently showed that all three scores perform equally well in predicting prospective all-cause mortality. However, Personal-MetaboHealth outperforms the other two scores in predicting time to first cardiometabolic disease and does so independently of smoking quantity, alcohol consumption, and medication use. A one standard deviation increase in the score is associated with a 13.69-year longer predicted median time until onset of the first cardiometabolic disease. Moreover, we showed that Personal-MetaboHealth can be improved by a combined lifestyle intervention (GOTO study), specifically in at risk individuals with unhealthy baseline scores. Together, these results establish Personal-MetaboHealth as a promising health check for individualised risk stratification and response to lifestyle interventions and therefore as a potential tool to guide preventive strategies in population health.

The original MetaboHealth score was created using metabolic biomarkers that were z-scaled separately per study population [7]. This was needed because the 12 used studies could not be analysed together, while we wanted to perform a direct comparison between metabolic biomarkers measured on different scales and ranges, facilitating feature selection based on effect size, and ensure that no single feature dominated the construction of the score. However, this means original MetaboHealth scores cannot be directly compared between individuals from different populations, limiting its usability. To overcome this problem, we scaled the metabolic biomarkers using a Dutch reference population (BBMRI-NL) consisting of 29,792 participants from 24 cohorts with age ranges from 16–103 years. This individualisation of the MetaboHealth score now allows direct comparisons of effects on health-related outcomes between individuals from different populations.

Over the last couple of years, Nightingale Health has clinically validated 39 of their metabolic biomarkers [14]. The advantage of restricting the Personal-MetaboHealth score to the 10 clinically validated metabolic biomarkers that were part of the original MetaboHealth score is that this would make the score more attractive for use in population health and clinical settings, because it would meet all regulatory requirements. Interestingly, the Personal-MetaboHealth outperformed the original MetaboHealth score in predicting cardiometabolic disease incidence, which we hypothesise to be partly due to the removal of metabolic biomarkers with more technical variability, such as acetoacetate and S-HDL-L [21]. Future studies will have to show if this is something specific for cardiometabolic disease or if the same applies to other health-related outcomes.

We quantified the effects of Personal-MetaboHealth as a change in hazard as well as on the age scale (using an AFT model). The advantage of the latter is that it provides a more intuitive interpretation that can be coupled back to an individual. These analyses showed a substantial and similar effect of Personal-MetaboHealth on both all-cause mortality (33% change in risk per change in standard deviation) and cardiometabolic disease onset (35% change in risk, or 13.69-year longer predicted median time until onset of the first cardiometabolic disease, per unit change in Personal-MetaboHealth), of which the magnitude is comparable to well-known risk factors such as socioeconomic status and smoking [25, 26]. Interestingly, the association between the original MetaboHealth score and cardiometabolic disease incidence seems to be largely explained by the effects of smoking, which is not surprising given the previously reported effects of smoking on both MetaboHealth [22] and, especially, cardiovascular disease [27]. Further research is needed to determine whether smoking acts primarily as a confounder, mediator, or collider in this association, or whether the observed attenuation mainly reflects shared variance between smoking and MetaboHealth. However, the association between Personal-MetaboHealth and cardiometabolic disease incidence is largely independent of smoking, indicating that this new score captures broader aspects of overall health than those affected by smoking.

Evidence regarding the effectiveness of lifestyle interventions in older adult populations remains inconclusive [28]. Therefore, we previously performed the GOTO study and showed that this combined diet and exercise intervention is able to improve overall health in individuals aged 46–75 years [27]. However, we observed a large heterogeneity in the response of individuals to the intervention, which was partly explained by difference in physical behaviour during the intervention [29]. In the current study, we found that another part of the heterogeneity in response may be due to differences in Personal-MetaboHealth scores at baseline. We showed that individuals with unhealthy Personal-MetaboHealth scores were able to improve their score during the intervention, which was coupled to improvements in other health parameters, such as blood pressure and markers of cholesterol metabolism. This suggests that the Personal-MetaboHealth score may be useful for identification and monitoring of at-risk middle-aged adults in need of a lifestyle intervention. We also observed that individuals with healthy Personal-MetaboHealth scores at baseline showed, on average, a slight decrease in their score during the intervention. However, the far majority of these individuals (67/83, 81%) retain a healthy score after the intervention. Moreover, these individuals still improved in many other health parameters, such as blood pressure, body composition, and markers of fat metabolism, during the intervention. More importantly, they did not show significant worsening in any of the tested health parameters. Hence, this indicates that the observed decrease in Personal-MetaboHealth likely represents fluctuations within the healthy range.

A strong focus in the field of biomarkers of ageing has been on CpG methylation-based scores, of which the mortality predictor GrimAge showed the most robust effects within the GOTO study [30]. Although the intervention effect on Personal-MetaboHealth only becomes apparent after stratifying individuals based on their baseline values, GrimAge declines in all individuals. While the decline in GrimAge is most strongly associated with a change in parameters related to body composition [30], we observed that the changes in individuals with unhealthy Personal-MetaboHealth scores seems to represent broader immune-metabolic changes, especially in fat metabolism and inflammation [30]. This indicates that metabolomics- and CpG methylation-based mortality predictors seem to capture different aspects of intervention-based health improvements and should ideally be used in combination.

A key strength of our study is the use of the prospective design of the LLS, with follow up for all-cause mortality of up to 22 years and cardiometabolic disease onset of up to 16 years. Detailed information on disease incidence and medication use was obtained directly from participants’ pharmacies and general practitioners, ensuring reliable outcome ascertainment. However, a limitation of the LLS is that the study design is complex due to its focus on families and the presence of right censoring and left truncation. Currently, no statistical method adequately accounts for both familial correlation and left truncation when estimating AFT models. This may impact the baseline hazards of the AFT models and thereby complicate interpretation on the age scale. Additionally, metabolomic data was only assessed at baseline, preventing the modelling of temporal changes. These limitations narrow the scope of inference but also highlight priorities for future research. Replication in larger, more diverse cohorts and the inclusion of longitudinal metabolomic trajectories will be essential to validate and extend these findings, but this was out of scope for the current study. Another limitation is that the scaling of Rescaled-MetaboHealth and Personal-MetaboHealth was based on BBMRI-NL, which is a reference population that mainly consists of Caucasian individuals and may thus provide an incomplete representation of the metabolomic diversity in other countries and ethnic populations, potentially, even within the Netherlands. As a next step we therefore plan to test the validity of the Personal-MetaboHealth in populations that are currently underrepresented within BBMRI-NL. Moreover, future studies can use a similar approach to create country-specific Personal-MetaboHealth variations based on large reference populations with Nightingale Health metabolomics data.

## Conclusions

In summary, we created an individualised version of the MetaboHealth score, which we termed Personal-MetaboHealth. In comparison to the original score, Personal-MetaboHealth retains its robust association with all-cause mortality in the LLS, while it shows an even stronger association with cardiometabolic disease onset, which was independent from smoking quantity, alcohol consumption, and medication use. Moreover, using data from the GOTO study, we found that Personal-MetaboHealth can be used to identify and monitor at-risk middle-aged individuals in need of a lifestyle intervention. Replication in larger and more diverse cohorts will be essential to confirm these associations and to establish applicability of Personal-MetaboHealth in different healthcare settings. Ultimately, integrating the Personal-MetaboHealth score into population health frameworks as an actionable health check in middle age may offer a powerful approach for early disease prevention and extension of healthy lifespan.

## Supplementary information

Supplementary Material 1: Additional file 1: Supplementary Methods. Figure S1, Tables S1-S3.

## Data Availability

The individual-level data from the LLS are protected by Dutch personal integrity laws, and other (privacy) regulations. As such, restrictions apply to the availability of the LLS data, which were used under license for the current study, and so are not publicly available. For both datasets, summary statistics are available upon request to the corresponding authors (Niels van den Berg and Joris Deelen). The LLS data is available following a data access procedure (https://leidenlangleven.nl/data-access/). Each request will be evaluated whether the research is compliant with the informed consent that has been signed by the LLS participants. The scripts containing the code for data pre-processing, data analyses, and MetaboHealth scores construction can be freely downloaded at https://git.lumc.nl/publications/individualized-metabohealth.

## Abbreviations

¹H-NMR: High-throughput nuclear magnetic resonance
AcAce: Acetoacetate
AFT: Accelerated failure time
Alb: Albumin
BBMRI-NL: Biobanking and BioMolecular Research Infrastructure Netherlands
CI: Confidence interval
Glc: Glucose
GOTO: Growing Old Together
GP: Glycoprotein acetyls
HR: Hazard ratio
His: Histidine
Ile: Iisoleucine
Lac: Lactate
Leu: Leucine
LLS: Leiden Longevity Study
Phe: Phenylalanine
PUFA/FA: The ratio of polyunsaturated fatty acids to total fatty acids
S-HDL-L: Total lipids in small HDL
Val: Valine
VLDL-D: Mean diameter for VLDL particles
XXL-VLDL-L: Total lipids in chylomicrons and extremely large VLDL
