# Supplementary material for: Personal-MetaboHealth, an actionable health check in middle age, is improved by an effective lifestyle intervention in those at risk
Source: BMC Med. 2026 Sep 7;24:470. doi: 10.1186/s12916-026-05178-z (PMC13548661; doi:10.1186/s12916-026-05178-z)
Supplement: Supplementary file 1 [file 12916_2026_5178_MOESM1_ESM.docx]

**Supplementary Information**

Personal-MetaboHealth, an actionable health check in middle age, is improved by an effective lifestyle intervention in those at risk

van den Berg et al.

**Supplementary Methods**

The standard approach that most researchers are familiar with consists of semi-parametric models that operate on the hazard scale. A well-known example is the Cox proportional hazards model. The baseline hazard describes how the risk of the outcome changes over time for an individual whose predictors are all set to their reference values (typically 0). In these models, the baseline hazard is estimated directly from the data, after which the model parameters are estimated conditional on the inferred baseline hazard. Another characteristic of Cox models is that they operate on the hazard scale. For example, when time is measured as the number of years after inclusion until an event occurs, this is translated into the instantaneous risk of experiencing the event during the subsequent time interval, namely the hazard and hazard ratio.

An Accelerated Failure Time model (AFT) model is a form of parametric survival analysis and therefore differs from Cox models on the aforementioned points, namely the baseline hazard and the interpretation of the time scale. The baseline hazard distribution must be specified a priori. Because the true underlying distribution is not directly observable, different candidate distributions are typically evaluated beforehand to determine which provides the best fit to the data. This is commonly assessed using Akaike (AIC) and Bayesian (BIC) information criteria by comparing otherwise identical models estimated under different distributional assumptions. Because the distribution is specified parametrically, the covariates can subsequently be modeled as a linear combination in relation to the outcome. As a result, conversion of the original time scale into a hazard distribution, as required in Cox models, is unnecessary. This is also why these models are referred to as Accelerated Failure Time models; the coefficients are interpreted not in terms of whether the risk of the outcome increases or decreases, but rather whether the outcome occurs faster or slower.

Specifying a baseline distribution a priori has the advantage that the original time scale can be retained, allowing for results that are generally more directly interpretable. A disadvantage, however, is that the estimates are sensitive to misspecification of the baseline distribution. It is therefore important to triangulate across different survival analysis methods, as we do by comparing Cox and AFT models. The AFT model for our analysis is specified as follows:

$$T_{j}=T_{0}\cdot\exp\left( Z_{j}\gamma+ C_{j}\alpha\right)\cdot exp(\sigma\varepsilon_{i})$$

Where $T_{j}$ represents the event time from inclusion to first cardiometabolic disease onset for individual j. $T_{0}$ reflects the baseline survival time and follows an exponential distribution. $Z_{j}$ is a vector of main exposure variables for individual *j*. The main exposure set consists of Personal-MetaboHealth. $C_{j}$ is the vector of covariates consisting of (1) sex and (2) age at inclusion. $\alpha$ is a vector of regression coefficients for the covariates. The main effects are on the log scale and *exp()* represents them as Time Ratios (TRs). *σ* is a scale parameter and represents variability of log-survival times around the linear predictor and $\varepsilon_{i}$ the error term for individual *i*, representing unexplained variation in survival time not accounted for by the covariates. Robust standard errors are used to account for family resemblance.

**Figure S1.** Distribution of the Personal-MetaboHealth score at baseline in GOTO study participants

**Table S1.** Association between the different MetaboHealth scores and all-cause mortality and cardiometabolic disease incidence adjusted for smoking

|  | **HR (95% CI)** | **P-value** | **HR (95% CI)** | **P-value** |
| --- | --- | --- | --- | --- |
| **Mortality** | **Basic**  **(*N* = 2,301)** | | **Smoking adjusted**  **(*N* = 1,871)** | |
| MetaboHealth | 0.77 (0.71 - 0.83) | 4.58 x 10^-10^ | 0.84 (0.76 - 0.93) | 5.25 x 10^-4^ |
| MetaboHealth-NL | 0.76 (0.70 - 0.83) | 2.24 x 10^-10^ | 0.82 (0.74 - 0.91) | 2.24 x 10^-4^ |
| Personal-MetaboHealth | 0.75 (0.69 - 0.82) | 5.31 x 10^-10^ | 0.83 (0.75 - 0.92) | 5.37 x 10^-4^ |
| **Cardiometabolic disease** | **Basic**  **(*N* = 1,347)** | | **Smoking adjusted**  **(*N* = 1,151)** | |
| MetaboHealth | 0.86 (0.78 - 0.95) | 0.003 | 0.89 (0.80 - 1.00) | 0.050 |
| MetaboHealth-NL | 0.86 (0.78 - 0.95) | 0.003 | 0.89 (0.80 - 1.00) | 0.046 |
| Personal-MetaboHealth | 0.74 (0.67 - 0.81) | 4.96 x 10^-10^ | 0.76 (0.68 - 0.85) | 1.21 x 10^-6^ |

HR, hazard ratio; 95% CI, 95% confidence interval. The basic models are adjusted for age at inclusion, sex, and family relations (random effect), while the smoking adjusted models are additionally adjusted for smoking.

**Table S2.** Changes in health markers in all GOTO participants and stratified by baseline Personal-MetaboHealth scores

|  | **All**  **(N = 135)** | | | | **Personal-MetaboHealth >= 0**  **(N = 83)** | | | | **Personal-MetaboHealth < 0**  **(N = 52)** | | | |
| --- | --- | --- | --- | --- | --- | --- | --- | --- | --- | --- | --- | --- |
| **Characteristics** | **Before** | **After** | **Change** | **P-value** | **Before** | **After** | **Change** | **P-value** | **Before** | **After** | **Change** | **P-value** |
| Age (years) | 62.82 | NA | NA | NA | 62.56 | NA | NA | NA | 63.24 | NA | NA | NA |
| Female (%) | 49.63 | NA | NA | NA | 50.60 | NA | NA | NA | 48.08 | NA | NA | NA |
| Systolic blood pressure (mmHg) | 138.80 | 135.84 | -2.96 | **0.002** | 137.00 | 133.66 | -3.34 | **0.004** | 141.66 | 139.30 | -2.36 | 0.172 |
| Diastolic blood pressure (mmHg) | 84.99 | 84.01 | -0.98 | 0.096 | 84.27 | 83.14 | -1.13 | 0.128 | 86.14 | 85.40 | -0.74 | 0.450 |
| BMI (kg/m2) | 26.83 | 25.63 | -1.19 | **<0.001** | 26.19 | 25.03 | -1.16 | **<0.001** | 27.84 | 26.59 | -1.26 | **<0.001** |
| Waist circumference (cm) | 96.11 | 91.48 | -4.63 | **<0.001** | 94.54 | 89.55 | -4.99 | **<0.001** | 98.62 | 94.56 | -4.06 | **<0.001** |
| Total body fat (%) | 32.16 | 30.65 | -1.69 | **<0.001** | 31.92 | 29.87 | -1.91 | **<0.001** | 32.58 | 31.83 | -1.33 | **<0.001** |
| Trunk fat (%) | 32.58 | 30.25 | -2.45 | **<0.001** | 31.57 | 28.69 | -2.70 | **<0.001** | 34.34 | 32.60 | -2.07 | **<0.001** |
| Fasting LDL cholesterol (mmol/L) | 2.39 | 2.23 | -0.16 | **<0.001** | 2.30 | 2.14 | -0.17 | **<0.001** | 2.52 | 2.37 | -0.15 | **0.001** |
| Fasting HDL cholesterol (mmol/L) | 1.50 | 1.51 | 0.01 | 0.322 | 1.58 | 1.58 | 0.00 | 0.861 | 1.37 | 1.40 | 0.04 | 0.051 |
| Fasting HDL size (nm) | 9.55 | 9.59 | 0.05 | **<0.001** | 9.60 | 9.65 | 0.04 | **<0.001** | 9.46 | 9.51 | 0.05 | **<0.001** |
| Fasting triglycerides (mmol/L) | 1.26 | 1.12 | -0.14 | **0.001** | 0.97 | 0.92 | -0.05 | 0.177 | 1.73 | 1.44 | -0.29 | **0.003** |
| Fasting insulin (mU/L) | 9.07 | 8.60 | -0.48 | 0.081 | 8.02 | 7.89 | -0.13 | 0.709 | 10.76 | 9.74 | -1.03 | **0.018** |
| Fasting CRP (mg/L) | 2.12 | 1.51 | -0.61 | **0.027** | 1.07 | 1.13 | 0.06 | 0.624 | 3.79 | 2.12 | -1.67 | **0.015** |

BMI, body mass index; CRP, C-reactive protein. P-values are based on the analyses of the Z-scaled health markers and adjusted for age at inclusion and sex at baseline as fixed effects (before intervention) and family-id and person-id as random effects. P-values <0.05 are highlighted in bold.

**Table S3.** Changes in Personal-MetaboHealth and the individual metabolic biomarkers contributing to the score in all GOTO participants and stratified by baseline Personal-MetaboHealth scores

|  | **All**  **(N = 135)** | | | | **Personal-MetaboHealth >= 0**  **(N = 83)** | | | | **Personal-MetaboHealth < 0**  **(N = 52)** | | | |
| --- | --- | --- | --- | --- | --- | --- | --- | --- | --- | --- | --- | --- |
| **Characteristics** | **Before** | **After** | **Change** | **P-value** | **Before** | **After** | **Change** | **P-value** | **Before** | **After** | **Change** | **P-value** |
| Personal-MetaboHealth | 0.20 | 0.22 | 0.02 | 0.666 | 0.66 | 0.53 | -0.13 | **0.007** | -0.54 | -0.28 | 0.26 | **0.003** |
| PUFA/FA (%) | 42.49 | 42.65 | 0.16 | 0.445 | 43.76 | 43.62 | -0.14 | 0.447 | 40.46 | 41.09 | 0.63 | 0.142 |
| Glycoprotein acetyls (mmol/L) | 0.83 | 0.80 | -0.02 | **<0.001** | 0.77 | 0.77 | 0.00 | 0.946 | 0.93 | 0.87 | -0.06 | **<0.001** |
| Glucose (mmol/L) | 5.27 | 5.17 | -0.10 | **0.001** | 5.16 | 5.07 | -0.09 | 0.053 | 5.46 | 5.33 | -0.13 | **0.002** |
| Albumin (g/L) | 40.01 | 39.65 | -0.36 | 0.058 | 40.30 | 39.77 | -0.53 | **0.035** | 39.56 | 39.47 | -0.08 | 0.768 |
| Phenylalanine (mmol/L) | 0.08 | 0.08 | 0.00 | 0.875 | 0.08 | 0.08 | 0.00 | **0.040** | 0.09 | 0.08 | 0.00 | **0.026** |
| Isoleucine (mmol/L) | 0.06 | 0.06 | 0.00 | 0.058 | 0.06 | 0.06 | 0.00 | 0.556 | 0.07 | 0.06 | 0.00 | **0.015** |
| Leucine (mmol/L) | 0.14 | 0.13 | -0.01 | **0.003** | 0.14 | 0.13 | 0.00 | 0.225 | 0.14 | 0.13 | -0.01 | **<0.001** |
| Valine (mmol/L) | 0.26 | 0.26 | 0.00 | 0.365 | 0.26 | 0.26 | 0.00 | 0.941 | 0.27 | 0.26 | -0.01 | 0.068 |
| Histidine (mmol/L) | 0.08 | 0.07 | -0.01 | **<0.001** | 0.08 | 0.07 | -0.01 | **<0.001** | 0.08 | 0.07 | 0.00 | **0.011** |
| Lactate (mmol/L) | 2.45 | 2.44 | -0.01 | 0.895 | 2.38 | 2.40 | 0.02 | 0.736 | 2.56 | 2.51 | -0.05 | 0.554 |

PUFA/FA, ratio of polyunsaturated fatty acids to total fatty acids. P-values are based on the analyses of the Z-scaled metabolic biomarkers and adjusted for age at inclusion and sex at baseline as fixed effects (before intervention) and family-id and person-id as random effects.
